# A novel pace-mapping technique for Isthmus Identification in Non-Sustained Uncommon Atrial Flutter Pacing Technique: the INPACT method—a case report

**DOI:** 10.1093/ehjcr/ytag139

**Published:** 2026-03-03

**Authors:** Daisuke Munakata, Takeshi Ueyama, Tomoyuki Uchida, Yasuhiro Ikeda

**Affiliations:** Department of Clinical Engineering, Yamaguchi Prefectural Grand Medical Center, 10077 Osaki, Hofu, Yamaguchi, 747-8511, Japan; Department of Cardiology, Yamaguchi Prefectural Grand Medical Center, 10077 Osaki, Hofu, Yamaguchi, 747-8511, Japan; Department of Cardiology, Yamaguchi Prefectural Grand Medical Center, 10077 Osaki, Hofu, Yamaguchi, 747-8511, Japan; Department of Cardiology, Yamaguchi Prefectural Grand Medical Center, 10077 Osaki, Hofu, Yamaguchi, 747-8511, Japan

**Keywords:** Macro-re-entry, Mapping technique, Localized re-entry, Ablation, iPASO, Case report

## Abstract

**Background:**

Mapping uncommon atrial flutter (AFL) remains challenging when tachycardia cannot be sustained during the procedure. We developed a novel pace-mapping methodology for identifying critical isthmuses in non-sustained uncommon AFL circuits during sinus rhythm.

**Case summary:**

A 70-year-old male presented with persistent AFL (cycle length 370 ms) following mitral valve repair with concomitant surgical pulmonary vein isolation. During electrophysiological study, the tachycardia terminated spontaneously, precluding conventional mapping. We implemented the Isthmus Identification in Non-Sustained Uncommon Atrial Flutter PACing Technique (INPACT), combining Intracardiac Pattern-match ScOring (iPASO) technology with systematic pace-mapping during sinus rhythm. A novel isthmus ratio (IR) metric differentiated potential isthmus sites among those with high morphological correlation (iPASO ≥90%). The critical isthmus was identified within a low-voltage area adjacent to the atrial septotomy line. Radiofrequency ablation targeting the site with the highest IR value eliminated the tachycardia with no recurrence at 6-month follow-up.

**Discussion:**

The INPACT enables identification of critical isthmuses without requiring sustained tachycardia, offering a solution for challenging cases where conventional mapping is limited by non-sustained arrhythmia. This approach may improve procedural efficiency and outcomes in complex uncommon AFL.

Learning points

- The Isthmus Identification in Non-Sustained Uncommon Atrial Flutter PACing Technique identifies critical isthmuses in non-sustained atrial flutter by combining Intracardiac Pattern-match ScOring pattern-matching with pace-mapping during sinus rhythm.
- The isthmus ratio metric differentiates central isthmus sites from exit sites, with values closest to 0 indicating optimal ablation targets.

## Introduction

Identification of critical isthmuses in uncommon atrial flutter (AFL) remains challenging, particularly when tachycardia spontaneously terminates during mapping procedures. Conventional activation and entrainment mapping require sustained tachycardia, limiting their utility in non-sustained arrhythmias.^1^ Traditional pace-mapping approaches have demonstrated success in ventricular tachycardia mapping,^2–4^ while Intracardiac Pattern-match ScOring (iPASO) mapping has enhanced accuracy for focal arrhythmias.^5^ We developed Isthmus Identification in Non-Sustained Uncommon Atrial Flutter PACing Technique (INPACT), integrating pace-mapping principles with iPASO technology to identify isthmuses during sinus rhythm, eliminating reliance on sustained tachycardia.

## Summary figure

**Figure:**
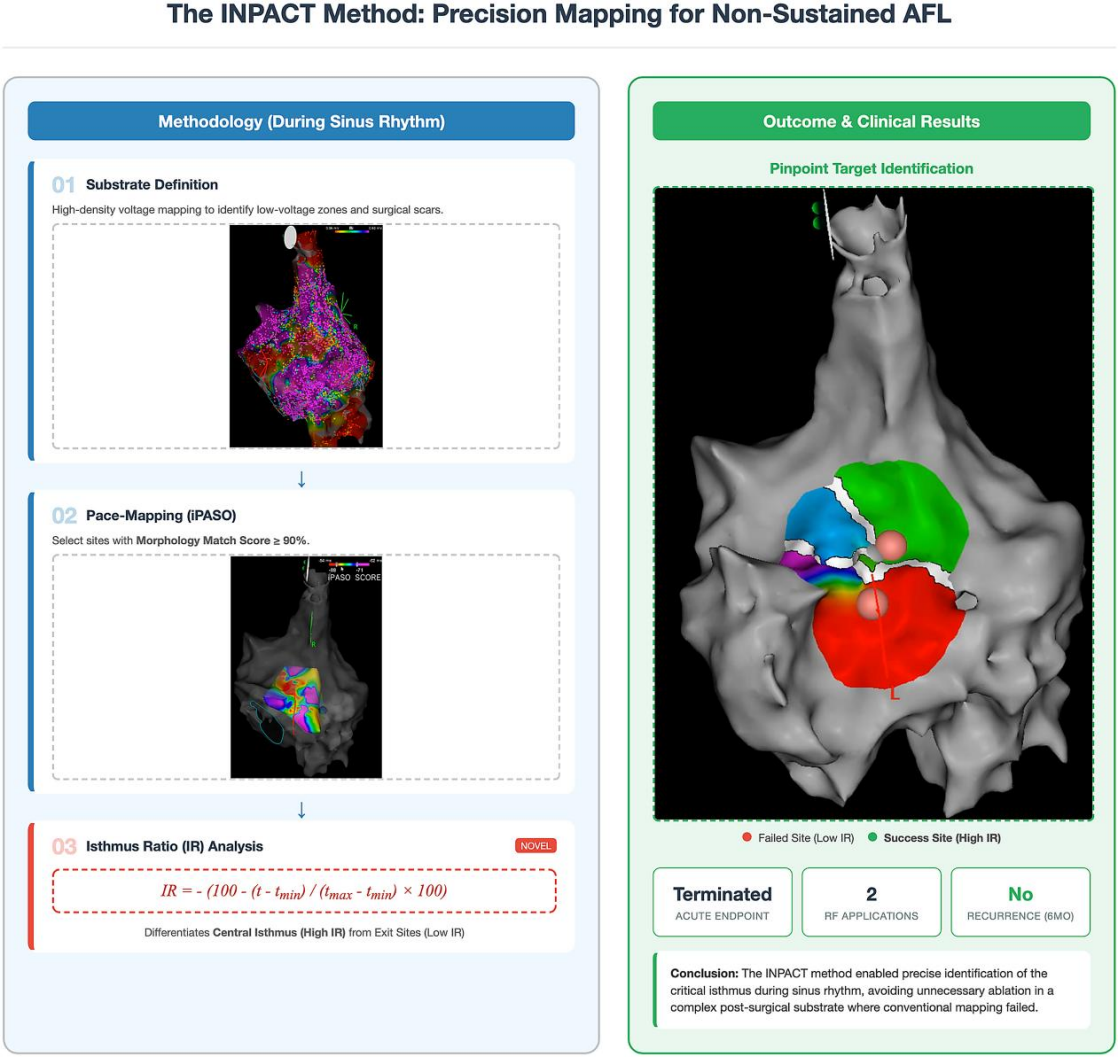
Overview of the Isthmus Identification in Non-Sustained Uncommon Atrial Flutter PACing Technique workflow and clinical outcome in non-sustained uncommon atrial flutter. During sinus rhythm, (1) high-density voltage mapping defines the substrate (low-voltage zones and post-surgical scar/incision lines), (2) systematic pace mapping with Intracardiac Pattern-match ScOring (iPASO) identifies candidate sites with a morphology match score ≥90%, and (3) the isthmus ratio (IR), calculated from the stimulus-to-distal coronary sinus time (*t*) among high-iPASO sites, differentiates exit sites (low IR) from the central critical isthmus (high IR). Isthmus Identification in Non-Sustained Uncommon Atrial Flutter PACing Technique-guided focal radiofrequency (RF) ablation at the high-IR target terminated the tachycardia, requiring two RF applications and showing no recurrence at 6-month follow-up (low-IR site: failed; high-IR site: successful).

## Case presentation

**Figure 1:**
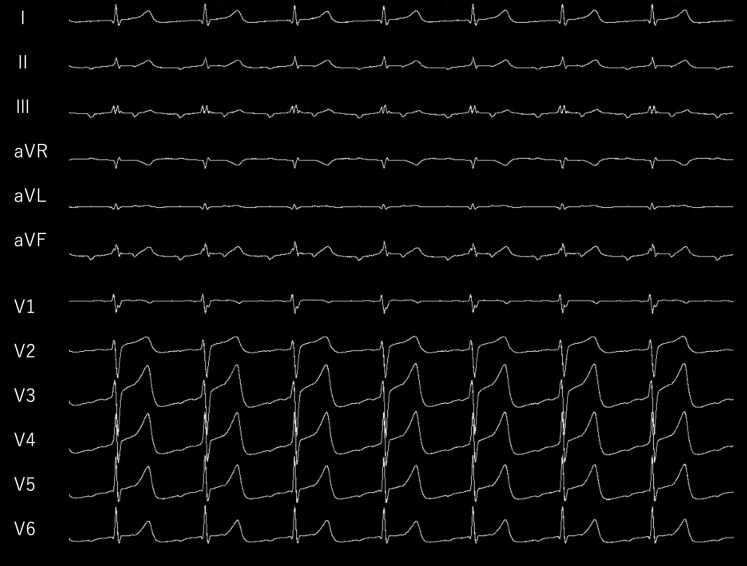
Twelve-lead ECG during the clinical tachycardia (uncommon atrial flutter; TCL = 370 ms).

A 70-year-old male was referred for catheter ablation of persistent AFL emerging after mitral valve repair with concomitant surgical pulmonary vein isolation. The patient presented with uncommon AFL exhibiting a cycle length of 370 ms (*Figure 1*). No antiarrhythmic drugs (Class I/III) were used before the procedure.

**Figure 2:**
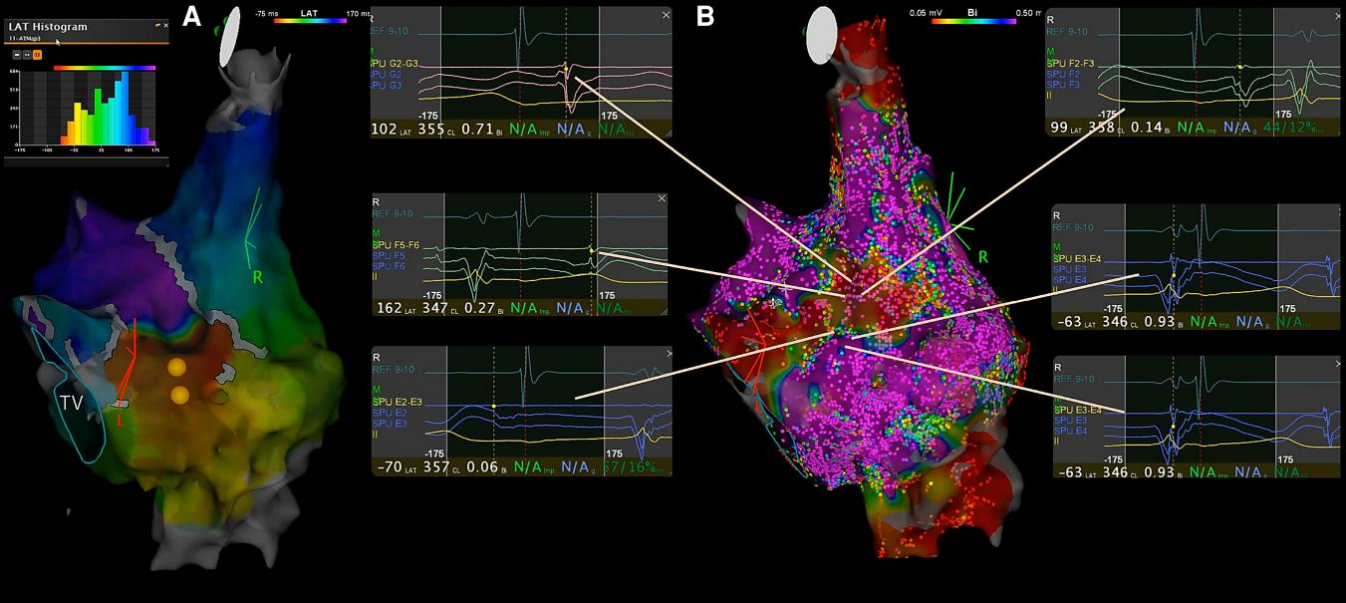
(*A*) Local activation time map during brief tachycardia. The histogram shows incomplete circuit mapping (Tachycardia Cycle Length = 370 ms). (*B*) Voltage map demonstrating low-voltage areas with fragmented electrograms near the atrial septotomy.

During electrophysiological assessment under general anaesthesia using CARTO 3 V8 (Biosense Webster, Diamond Bar, CA, USA), abnormal fractionated potentials were observed near the atrial septotomy margin. Post-pacing interval mapping during brief tachycardia episodes yielded values matching the tachycardia cycle length, suggesting a right atrial (RA) circuit. However, the ///LAT histogram did not demonstrate complete re-entry (*Figure 2A*), and sustained tachycardia could not be maintained.

We implemented the INPACT. High-density voltage mapping delineated low-voltage zones and the septotomy line (*Figure 2B*). Systematic pace-mapping was performed at 600 ms cycle length from multiple sites near the surgical incision and low-voltage areas. The Intracardiac Paced-Morphology Comparison module compared P-wave morphology from each paced site against the clinical tachycardia template using coronary sinus (CS) electrograms.^6^

Sites with iPASO scores ≥90% underwent further analysis using our novel IR:


$$\mathrm{IR}=-\left(100-\frac{t-t_{\min}}{t_{\max}-t_{\min}}\times 100\right)$$

where *t* represents stimulus-to-CS-distal time at the pacing site, with $t_{\min}$ and $t_{\max}$ being minimum and maximum times among high-iPASO sites. Lower IR values suggest exit sites, while higher values indicate central isthmus locations. Isthmus ratio is a normalized index of stimulus-to-distal CS time (*t*) among high-iPASO sites; thus shorter *t* indicates proximity to the exit (lower IR), whereas longer *t* suggests an upstream central isthmus location (higher IR). In this case, IR was calculated manually for 11 high-iPASO sites (∼10 min). Notably, the leading minus sign in the IR equation was intentionally introduced so that exit sites are displayed in red on the electroanatomical map (consistent with conventional activation mapping), and omitting this inversion would reverse the colour interpretation.

**Figure 3:**
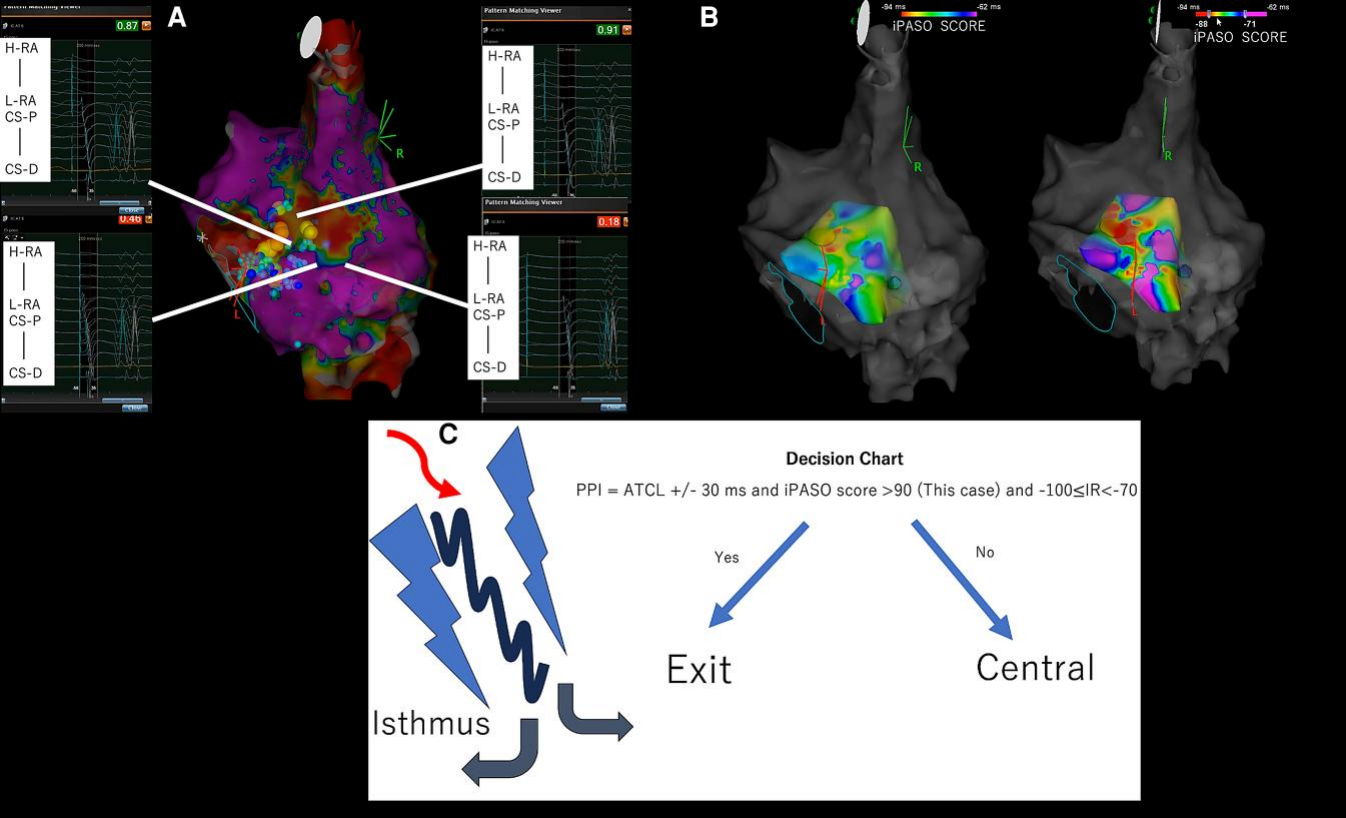
(*A*) Intracardiac Pattern-match ScOring score distribution: yellow (≥90%), blue (≥85%), and light blue (≥75%). High scores concentrate along the septal isthmus. (*B*) Alternative visualization with Intracardiac Pattern-match ScOring scores as negative values. (*C*) Decision algorithm integrating electrophysiological parameters for isthmus targeting.

**Figure 4:**
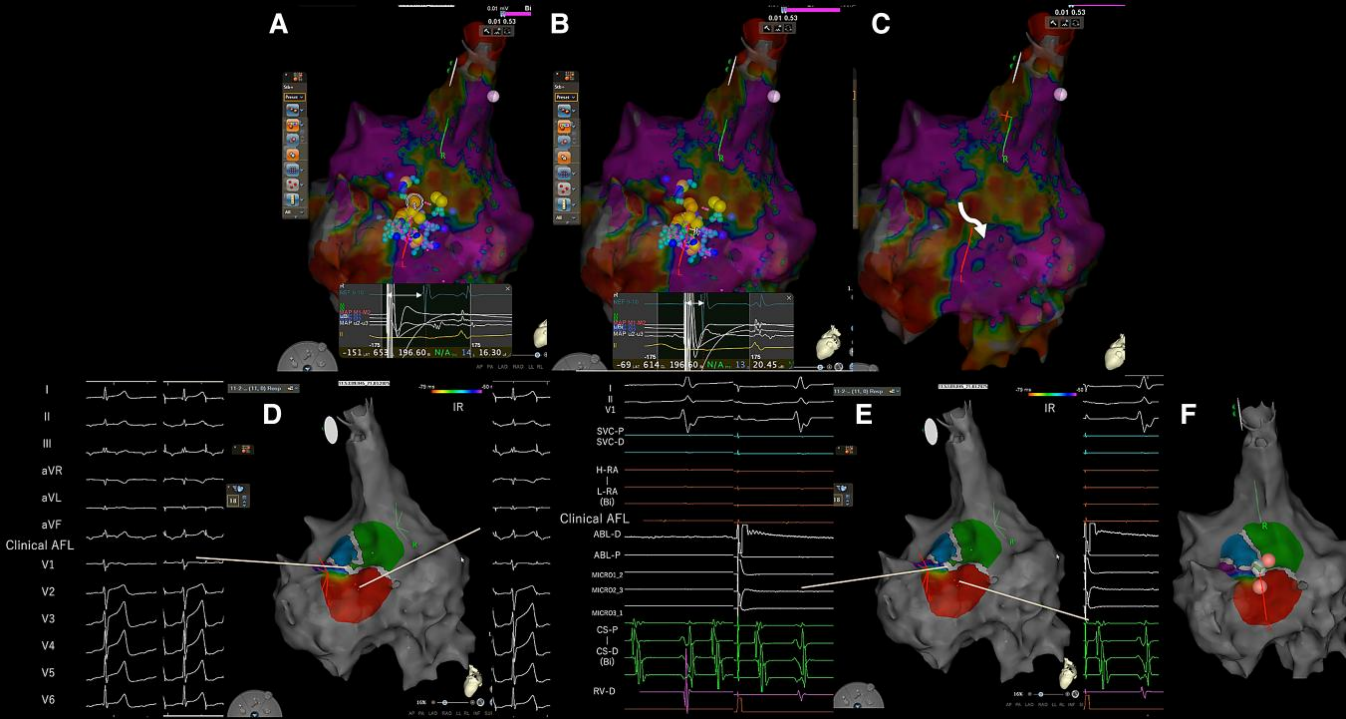
(*A–C*) Activation map colour-coded by isthmus ratio for high-Intracardiac Pattern-match ScOring sites. (*D*) Twelve-lead electrocardiogram during pacing from central isthmus showing morphology match with clinical tachycardia. (*E*) Intracardiac electrograms during isthmus pacing. (*F*) Isthmus Identification in Non-Sustained Uncommon Atrial Flutter PACing Technique-guided focal ablation sites. Red area: initial ablation at low isthmus ratio site (unsuccessful). Green area: successful ablation at high IR site (central isthmus).

The highest iPASO score (94%) localized to the RA septal wall within a low-voltage area adjacent to the septotomy line (*Figures 3 and 4A–C*). To facilitate visualization of the iPASO score distribution on the electroanatomical map, scores were also displayed as negative values (*Figure 3B*).^7^ Initial RF ablation at a presumed exit site (low IR value) failed to eliminate inducibility. Subsequent ablation at the site with the highest IR value (central isthmus) successfully rendered the tachycardia non-inducible with a single application. Post-ablation monitoring confirmed stable sinus rhythm with no recurrence at 6-month follow-up. No antiarrhythmic drugs (Class I/III) were prescribed after ablation during follow-up.

## Discussion

This case demonstrates the clinical utility of INPACT mapping for complex uncommon AFLs when sustained tachycardia cannot be maintained. By combining systematic pace-mapping with iPASO pattern-matching and the quantitative IR metric, critical isthmuses can be identified during sinus rhythm. This tachycardia-independent approach addresses a significant limitation of conventional techniques.^1^

The spatial distribution of iPASO scores provides mechanistic insights—the linear high-score pattern along the identified isthmus contrasts with concentric patterns in focal atrial tachycardias,^5^ potentially aiding in mechanism differentiation. The anatomical substrate of post-surgical scar likely contributes to this specificity.

### Ablation strategy

The INPACT method enabled a highly targeted ablation approach, achieving complete success with only two focal RF applications. This precision avoided unnecessary tissue ablation, particularly advantageous in post-surgical patients with existing atrial scarring. The minimal ablation strategy demonstrates that accurate isthmus localization can reduce procedure time and minimize collateral damage.

Key advantages include workflow efficiency by eliminating prolonged tachycardia induction requirements and the ability to corroborate findings with brief tachycardia episodes. The integration of established pace-mapping principles^2–4^ with modern pattern-matching technology represents a significant methodological advance. A combined pacing and pattern-matching approach has been reported for identifying critical isthmuses in re-entrant atrial tachycardia during sustained tachycardia.^8^

### Methodological considerations

While dual-chamber pattern matching may provide superior accuracy compared to CS-only referencing,^5^ we attempted dual-chamber recording but could not obtain adequate RA electrograms due to low voltage (*Figure 3A*). Despite this limitation, our CS-predominant approach successfully identified the tachycardia origin, validated by high P-wave morphology concordance (*Figure 4D*) and clinical success. This is supported by a feasibility report of atrial pace mapping using intracardiac pattern matching, suggesting region-dependent accuracy with lower performance at lateral sites but higher performance at anteroseptal sites.^9^ This is also supported by evidence demonstrating 91.2% accuracy for CS-based Intracardiac Pattern-Matching in discriminating RA pacing sites.^10^ Nevertheless, dual-chamber referencing should be pursued when feasible to maximize accuracy.

### Limitations

This single case requires validation in larger studies. Additional limitations include dependence on brief tachycardia episodes for template acquisition and challenges in vast substrates with multiple high-iPASO sites. The method's effectiveness for shorter cycle tachycardias requires investigation.

Future research should validate INPACT across diverse AFL mechanisms. Automated analysis algorithms may improve clinical accessibility.

## Lead author biography


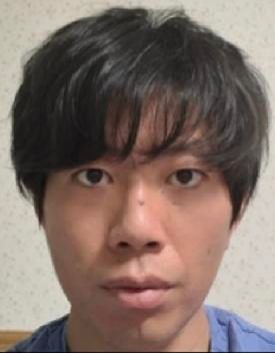


Daisuke Munakata obtained a Master of Science degree from Yamaguchi University in 2019. Since 2014, he has been working in the Clinical Engineering Department at Yamaguchi Prefectural Grand Medical Center. As a Clinical Engineer, he specializes in cardiac ablation procedures and electrophysiology support.

## Data Availability

The data underlying this article are available in the article. Additional anonymized patient data may be shared on reasonable request to the corresponding author with appropriate ethics approval.

## Notes

**Consent:** Written informed consent was obtained from the patient for publication of this case report and accompanying images in accordance with COPE guidelines.

## References

[1] Luther V, Sikkel M, Bennett N, Guerrero F, Leong K, Qureshi N, et al Visualizing localized reentry with ultra-high density mapping in iatrogenic atrial tachycardia: beware pseudo-reentry. Circ Arrhythm Electrophysiol 2017;10:e004724. 28356307 10.1161/CIRCEP.116.004724

[2] Brunckhorst CB, Delacretaz E, Soejima K, Maisel WH, Friedman PL, Stevenson WG. Identification of the ventricular tachycardia isthmus after infarction by pace mapping. Circulation 2004;110:652–659. 15289385 10.1161/01.CIR.0000138107.11518.AF

[3] Ellison K, Friedman P, Ganz L, Stevenson W. Entrainment mapping and radiofrequency catheter ablation of ventricular tachycardia in right ventricular dysplasia. J Am Coll Cardiol 1998;32:724–728. 9741518 10.1016/s0735-1097(98)00292-7

[4] de Chillou C, Groben L, Magnin-Poull I, Andronache M, MagdiAbbas M, Zhang N, et al Localizing the critical isthmus of postinfarct ventricular tachycardia: the value of pace-mapping during sinus rhythm. Heart Rhythm 2014;11:175–181. 24513915 10.1016/j.hrthm.2013.10.042

[5] Yamashita K, Furuya K, Sato Y, Kinebuchi Y, Funayama K, Masano T, et al Intracardiac electrogram-based atrial pace mapping for detecting the earliest activation site in atrial arrhythmias. Heart Rhythm 2024;21:1400–1408. 38369035 10.1016/j.hrthm.2024.02.028

[6] Hayashi K, Mathew S, Heeger C-H, Maurer T, Lemes C, Riedl J, et al Pace mapping for the identification of focal atrial tachycardia origin: a novel technique to map and ablate difficult-to-induce and nonsustained focal atrial tachycardia. Circ Arrhythm Electrophysiol 2016;9:e003930. 27390210 10.1161/CIRCEP.116.003930

[7] Nishiuchi S, Sato T, Yamagami S, Kondo H, Tamura T. Novel automated atrial pacemapping technique based on intracardiac pattern matching to identify the location of ectopic atrial activity. J Interv Card Electrophysiol 2024;67:671–674. 38649589 10.1007/s10840-024-01812-z

[8] Munakata D, Uchida T, Ueyama T, Ikeda Y. A novel combined pacing and pattern matching approach for identifying critical isthmuses in reentrant atrial tachycardia. HeartRhythm Case Rep 2025;11:567–571. 40557394 10.1016/j.hrcr.2025.03.020 PMC12184848

[9] Willert S, Demmig T, Maslova V, Zaman A, Popera J, Nicholson L, et al Atrial pace mapping using intracardiac pattern matching: feasibility and accuracy. Europace 2024;26:euae102.692.

[10] Guerra P, Yarnitsky J, Macle L, Sablayrolles Y, Lavoie C, Nakar E, et al Validation of an algorithm for automatic arrhythmia recognition and 3D mapping in a porcine model. Authorea 2022. 10.22541/au.164864960.09178374/v1

